# Classification of Cervical Spine Fracture and Dislocation Using Refined Pre-Trained Deep Model and Saliency Map

**DOI:** 10.3390/diagnostics13071273

**Published:** 2023-03-28

**Authors:** Soaad M. Naguib, Hanaa M. Hamza, Khalid M. Hosny, Mohammad K. Saleh, Mohamed A. Kassem

**Affiliations:** 1 Department of Information Systems, Faculty of Computers and Informatics, Zagazig University, Zagazig 44519, Egypt; 2 Department of Information Technology, Faculty of Computers and Informatics, Zagazig University, Zagazig 44519, Egypt; 3 Department of Orthopedic Surgery, Faculty of Medicine, Zagazig University, Zagazig 44519, Egypt; 4 Department of Robotics and Intelligent Machines, Faculty of Artificial Intelligence, Kafrelsheikh University, Kafrelsheikh 33516, Egypt

**Keywords:** deep learning, X-ray, cervical spine fractures, cervical spine dislocation, computer aided-diagnosis system

## Abstract

Cervical spine (CS) fractures or dislocations are medical emergencies that may lead to more serious consequences, such as significant functional disability, permanent paralysis, or even death. Therefore, diagnosing CS injuries should be conducted urgently without any delay. This paper proposes an accurate computer-aided-diagnosis system based on deep learning (AlexNet and GoogleNet) for classifying CS injuries as fractures or dislocations. The proposed system aims to support physicians in diagnosing CS injuries, especially in emergency services. We trained the model on a dataset containing 2009 X-ray images (530 CS dislocation, 772 CS fractures, and 707 normal images). The results show 99.56%, 99.33%, 99.67%, and 99.33% for accuracy, sensitivity, specificity, and precision, respectively. Finally, the saliency map has been used to measure the spatial support of a specific class inside an image. This work targets both research and clinical purposes. The designed software could be installed on the imaging devices where the CS images are captured. Then, the captured CS image is used as an input image where the designed code makes a clinical decision in emergencies.

## 1. Introduction

**Figure 1:**
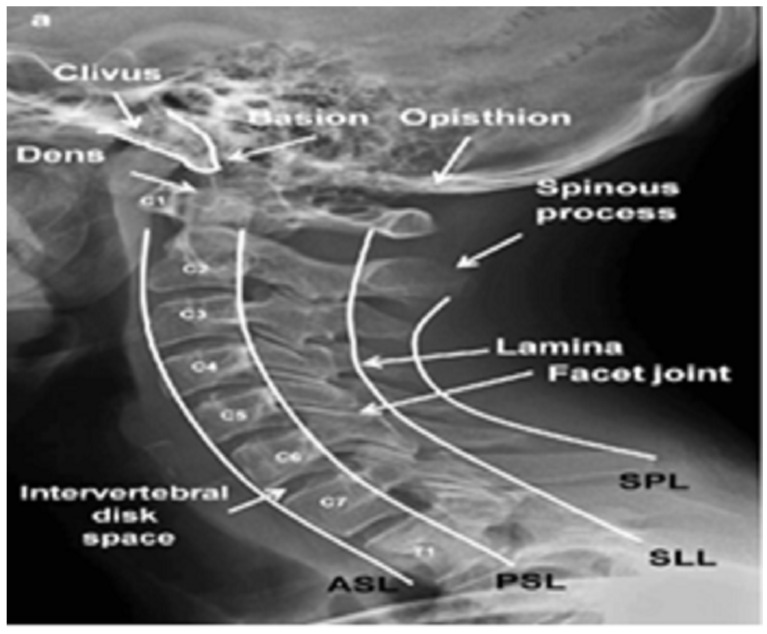
Lateral view of the CS [3].

The first seven vertebrae in the spine below the skull and above the thoracic spine are known as CS. The CS is divided into two groups that are both anatomically and functionally distinct: the upper pair (C1 and C2), the axis and atlas, respectively, and the lower five (C3–C7), the subaxial cervical vertebrae [1,2] as shown in Figure 1.

The CS injury rate is high and significantly impacts human life [4,5,6]. It comprises bony and soft structures and plays several critical biological roles in the body’s functioning, supporting the head and blood supply to the brain and protecting the spinal cord. Unfortunately, it is susceptible to injury, degeneration, aging, and disease. 

Recently, statistics from the United States [7] show that 38.2%, 32.3%, 14.3%, 7.8%, 4.1%, and 3.3% are the percentages of CS injury by car accidents, falls, violence, sports, and recreational activities, medical errors, and other factors, respectively, where severe injuries can cause fractures and/or dislocations and consequently lead to more serious consequences such as significant functional disability, or even death, which results in a large financial burden [8,9,10,11]. 

Therefore, diagnosing CS injuries should be done urgently without any delay. The first step in evaluating polytraumatized patients is screening the CS through imaging [12]. The early detection of CS injuries in emergency units could be performed using the X-ray imaging modality. X-ray imaging is one of the earliest, most popular, and cheap methods used in clinical medicine, which produces images of any bone, including the hand, hip, wrist, hip pelvis, CS, etc., to detect bone fractures. 

Physicians and radiologists must visually inspect many X-ray images to identify the injured cases of CS, which may lead to diagnostic errors. Statistically, 80% of diagnostic errors in emergency units are caused by false interpretations by physicians who lack the required specialized expertise or due to exposure to fatigue during a busy day [13]. Therefore, building an accurate computer-aided-diagnosis system based on AI to support physicians in interpreting CS X-ray images can decrease errors, reduce physicians’ stress, and improve medical service quality. 

AI is a very powerful technology in radiographic image interpretation [14,15,16]. Using AI in radiology practices is beneficial in healthcare [17]. It has been estimated that using AI for automated image interpretation could reduce the time spent by radiologists reviewing images by 20% [18]. Various studies have been conducted to evaluate the performance of AI in detecting fractures. For example, [19] developed and tested a deep-learning system to provide clinicians with the timely fracture detection expertise of experts in musculoskeletal imaging. They developed a deep-learning system for detecting fractures across the musculoskeletal system, trained it on data manually annotated by senior orthopedic surgeons and radiologists, and then evaluated its ability to emulate them. 

The overall AUC of the deep-learning system was 0.974 (95% CI: 0.971–0.977), sensitivity was 95.2% (95% CI: 94.2–96.0%), specificity was 81.3% (95% CI: 80.7–81.9%), positive predictive value (PPV) was 47.4% (95% CI: 46.0–48.9%), and negative predictive value (NPV) of 99.0% (95% CI: 98.8–99.1%). Secondary tests of the radiographs with no inter-annotator disagreement yielded an overall AUC of 0.993 (95% CI: 0.991–0.994), a sensitivity of 98.2% (95% CI: 97.5–98.7%), specificity of 83.5% (95% CI: 82.9–84.1%), PPV of 46.9% (95% CI: 45.4–48.5%), and NPV of 99.7% (95% CI: 99.6–99.8%). 

Hardalaç and his co-authors conducted another study [20] to perform fracture detection using deep learning on wrist X-ray images to support physicians in diagnosing wrist fractures, particularly in emergency services. Using SABL, RegNet, RetinaNet, PAA, Libra R-CNN, FSAF, faster R-CNN, dynamic R-CNN, and DCN deep-learning-based object detection models with various backbones, 20 different fracture detection procedures were performed on a dataset of wrist X-ray images. Five different ensemble models were developed and then used to reform an ensemble model to develop a unique detection model, ‘wrist fracture detection-combo (WFD-C)’, to further improve these procedures. From 26 different models for fracture detection, the highest detection result obtained was 0.8639 average precision (AP50) in the WFD-C model. 

In addition, in [21], a universal fracture detection CAD system was developed on X-ray images based on the deep learning method. Firstly, we design an image preprocessing method to improve the poor quality of these X-ray images and employ several data augmentation strategies to enlarge the used dataset. We propose our automatic fracture detection system based on our modified Ada-ResNeSt backbone network and the AC-BiFPN detection method. Finally, we established a private universal fracture detection dataset MURA-D based on the public dataset MURA. 

As demonstrated by our comprehensive experiments, compared with other popular detectors, our method achieved a higher detection AP of 68.4% with an acceptable inference speed of 122 ms per image on the MURA-D test set, achieving promising results among state-of-the-art detectors.

Despite the wide utilization of AI in medicine, few methods have been proposed for detecting fractures in the CS. Salehinejad et al. [22] proposed a deep convolutional neural network (DCNN) with a bidirectional long short-term memory (BLSTM) layer for the automated detection of cervical spine fractures in CT axial images. They used an annotated dataset of 3666 CT scans (729 positives and 2937 negative cases) to train and validate the model. 

The validation results show a classification accuracy of 70.92% and 79.18% on the balanced (104 positive and 104 negative cases) and imbalanced (104 positives and 419 negative cases) test datasets. In addition, in a study by [12], 665 examinations were included in their analysis. The k coefficients, sensitivity, specificity, and positive and negative predictive values were calculated with 95% CIs comparing the diagnostic accuracy and agreement of the convolutional neural network and radiologist ratings, respectively, compared with ground truth. 

The obtained results showed that convolutional neural network accuracy in the CS fracture detection was 92% (95% CI, 90–94%), with 76% (95% CI, 68–83%) sensitivity and 97% (95% CI, 95–98%) specificity. The radiologist’s accuracy was 95% (95% CI, 94–97%), with 93% (95% CI, 88–97%) sensitivity, and 96% (95% CI, 94–98%) specificity. Fractures missed by the convolutional neural network and by radiologists were similar by level and location and included fractured anterior osteophytes, transverse processes, and spinous processes, as well as lower cervical spine fractures that are often obscured by CT beam attenuation. 

Based on previous studies, no published paper/study used AI to detect the fracture and/or dislocation on X-ray CS. All previous studies only identified a CS fracture, which motivates the authors to present a new CNN-based model to detect fracture and dislocation in the X-images of CS, as the fractures may be only minor injuries. However, the dislocation of the CS may be a life-threatening condition [6]. Unfortunately, the dataset of the previously mentioned studies is not available. 

The proposed model is an efficient pre-trained model, classifying the X-images of CS into three classes (normal, fracture, and dislocation) with an accuracy of 99%. The proposed system aims to support physicians in diagnosing CS injuries, especially in emergency units. The main contributions of the proposed model are:

1. Using X-ray images, a new pre-trained CNN-based model for detecting fractures and dislocation in the CS.
2. The proposed model is efficient, easily installed, and executed using regular configuration PC machines or low-cost embedded systems.
3. The proposed model successfully classified unlabeled 68 X-ray images of CS.
4. The ability of the proposed model to successfully detect the cervical spine was proven by the saliency map.

## 2. Materials and Methods

With rapidly increasing applications in atomistic, image-based, spectral, and textual data modalities, deep learning (DL) is one of the areas of data science that is rapidly expanding. Artificial intelligence (AI) uses machine-learning algorithms called DCNNs, frequently used to identify medical images. Data analysis and feature identification are made possible by DL. The basic idea is to feed each layer of the neural network with the pixel values from a digital image using methods such as convolution and pooling, and then update the weights in the neural network based on how different the output is from the real label. The weights in the neural network are changed to meet the problem once a sizable amount of imaging input is used as the training data. A detailed description of the deep-learning techniques utilized in this study is provided in the following subsection, followed by a thorough analysis of the experiments.

### 2.1. Deep Neural Networks

Recently, deep neural networks have been widely used as a potentially effective alternative feature extraction method [23,24,25]. These networks automatically choose the most crucial features [26]. This approach is a powerful development in AI [27]. The following subsections will briefly describe the convolutional neural networks (CNNs). The CNN is a stack of layers that learns something from the layer before it, either linearly or nonlinearly [28,29,30]. Any CNN model includes several layers, such as the convolutional layer, ReLU (activation function), batch normalization (a technique to reduce distribution drift by each mini-batch), pooling layer, etc. Each of the preceding layers has the main task of filtering and extracting features, swapping out all negative pixel values for zero in the feature map, reducing the feature map dimensions by keeping the most crucial feature, and normalizing input data across all batches for each channel, respectively. 

Transfer learning is a strategy that reuses a trained model to execute one task in another. It is typically utilized in cases of insufficient training data [31]. In transfer learning, deep neural networks are learned using big datasets where the model weights are saved [32,33]. Therefore, deep transfer learning on a large dataset can help fine-tune a pre-trained model with small datasets. In addition, it enables handling the classification of both binary and multi-class. Furthermore, transfer learning is more effective in diagnosing dislocation and fracture, making it the preferred method.

**Figure 2:**
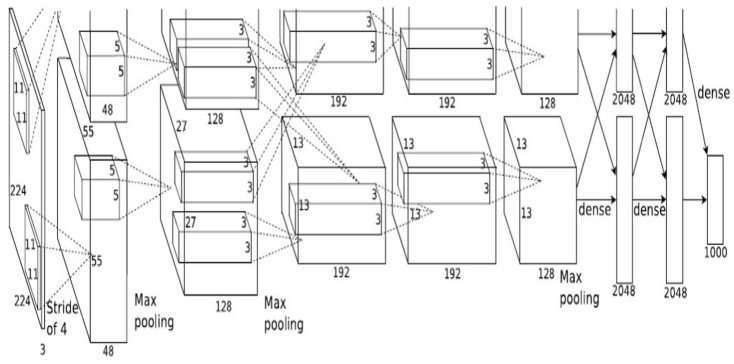
AlexNet architecture [34].

We tested the proposed method with high-performance measures using real-spine dislocation, fracture, and spine-normal cases. We utilized AlexNet and GoogleNet in this work, using the saved weights. We re-trained this net using our dataset to enable the network layers to differentiate between spine dislocation, spine fracture, and spine-normal with high precision. We used this transfer learning network to train the models on the spine dataset and then examined the predictions. One of the most well-known neural network designs to date is AlexNet. It is based on convolutional neural networks and was suggested by Alex Krizhevsky for the ImageNet Large-Scale Visual Recognition Challenge (ILSVRV) [34]. The architecture has eight layers, the first five of which are convolutional layers and the final three of which are completely connected. The first two convolutional layers are linked to overlapping max-pooling layers to obtain the maximum possible features. The fully connected layers are immediately connected to the third, fourth, and fifth convolutional layers. The ReLu nonlinear activation function is linked to each output of the convolutional and fully connected layers. A SoftMax activation layer, which generates a distribution of 1000 class labels, is linked to the final output layer. The network contains over 60 million parameters and 650,000 neurons. The network employs dropout layers to minimize overfitting during the training phase. The “dropped out” neurons do not add to the forward pass or engage in backpropagation. The first two fully connected levels contain these sections. The full architecture of AlexNet is shown in Figure 2.

**Figure 3:**
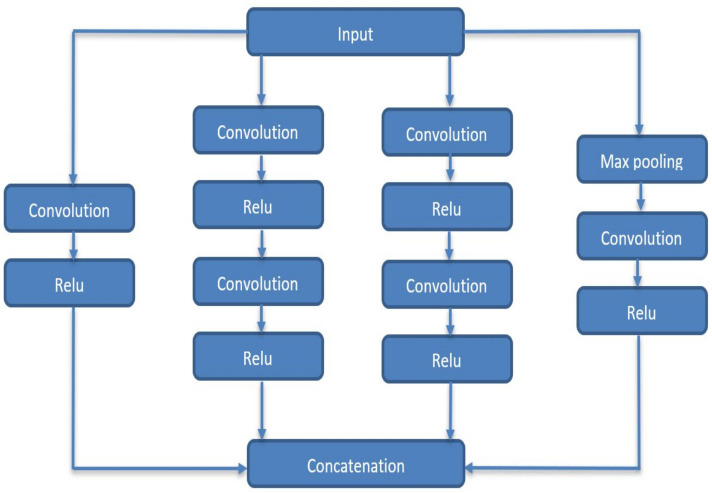
Inception module in GoogleNet.

The GoogleNet architecture was created with a sparse connection between activations. In other words, not all 512 output channels will be connected to all 512 input channels using pruning strategies. Therefore, GoogleNet is an inception module [35] approximating a sparse CNN with conventional dense architecture. GoogleNets employs convolutions of various sizes (5 × 5, 3 × 3, 1 × 1) to capture information at various scales (Figure 3). 

As an example, consider GoogleNet’s initial conception module. It has 192 input channels. It only includes a 128 kernel size 3 × 3 filters and 32 sizes 5 × 5 filters. For 5 × 5 filters, it requires (25 × 32 × 192) where the number of 5 × 5 filters can be further increased. The inception module employs small convolutions (1 × 1) before bigger-sized kernels to reduce input channel dimensions, which are then fed into those convolutions. Additionally, in GoogleNet, after the last convolutional layer, the fully connected layer is replaced with a simple global average pooling layer to average the channel values throughout a 2D feature map. Consequently, the overall number of parameters is considerably reduced compared with AlexNet, where the fully connected layer holds most parameters (+90%). 

### 2.2. The Proposed Algorithm

This section explains the classification procedure for CS (dislocation, fracture, and normal). The dataset’s primary flaw is the degree of similarity across various cervical spine types (dislocation, fracture, and normal). Therefore, for this task, we require an accurate computer-aided-diagnosis system.

#### 2.2.1. Preprocessing

We carried out image preprocessing after the image acquisition. The dataset images are in the grayscale color space with different widths and height sizes. The AlexNet and GoogleNet input channels had three data channels corresponding to the three colors (red, green, and blue). In contrast, the input image height and width of the input image must be (227 × 227) for AlexNet or (224 × 224) for GoogleNet. Image preprocessing involves two processes. The first step is resizing all images to match the AlexNet or GoogleNet input layer. The second is duplicating the original image three times to overcome the issue of the input channel (R, G, B), as explained in the following Algorithm 1.
**Algorithm 1** Duplication image for input channelsInput: one-channel imageOutput: Three-channel image1  I = read the image2  C = number of channels for (I)3     If input image (C,1)4     Concatenate arrays along specified dimensions (I, I, I)5    Repeat step 1 to 4 for all images in the dataset6     END7  END

#### 2.2.2. Feature Extraction

Feature extraction is the most important stage in classification. A pre-trained transfer learning model was used to extract features, identify an image’s key aspects, and extract information from them. A model is created by stacking many CNNs back-to-back. We utilized two pre-trained models called AlexNet and GoogleNet in this case. It is a multidimensional version of the logistic function used in multinomial logistic regression. The ImageNet weights were utilized in the pre-trained net. The bottom levels were replaced with a fully connected layer that combines the data extracted by the preceding layers to generate the final output and a SoftMax layer that turns a vector of K-real values into a probability distribution with K-potential outcomes. The final activation function is the SoftMax function, frequently used to normalize the network output to a probability distribution over the predicted output class.

#### 2.2.3. Classification

**Figure 4:**
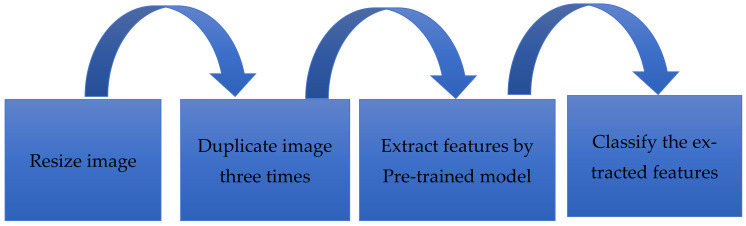
The overall process of the proposed method.

**Figure 5:**
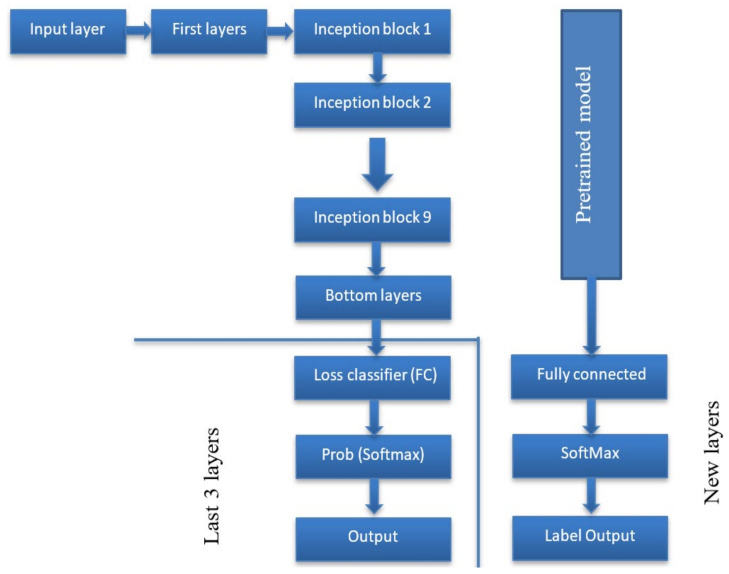
The architecture of the proposed method.

A classification layer is the last layer which derives the number of classes from the preceding layer’s output size. The extracted features were fed to the classification layer. This layer returns a three-value array indicating each diagnosis group’s probability. The class number corresponded to three distinct CSs. The class numbers are assigned for c-spine_dislocation (0), c-spine_fracture (1), and c-spine_normal (2). The overall process and architecture of the proposed method are shown in Figure 4 and Figure 5, respectively.

## 3. Experiments

The experiments were performed using an IBM-compatible machine outfitted with a Core i7-CPU, 16GB-DDRAM, and a GeForce MX150 NVIDIA graphics card. The application was run on an x64-bit MATLAB 2018. The maximum number of training epochs was set to 30, with a mini-batch size of 10, the starting learning rate was set at 0.001, and the momentum was set to 0.95.

### 3.1. Datasets

**Figure 6:**
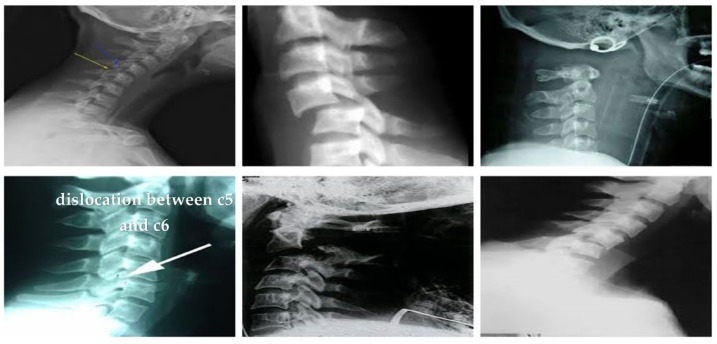
Samples from the first group (CS dislocation images).

**Figure 7:**
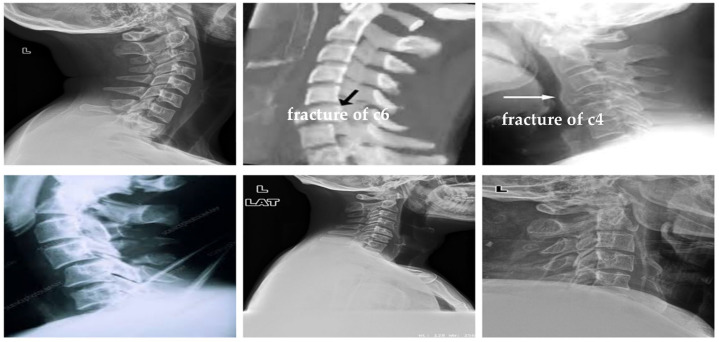
Samples from the second group (CS fracture images).

**Figure 8:**
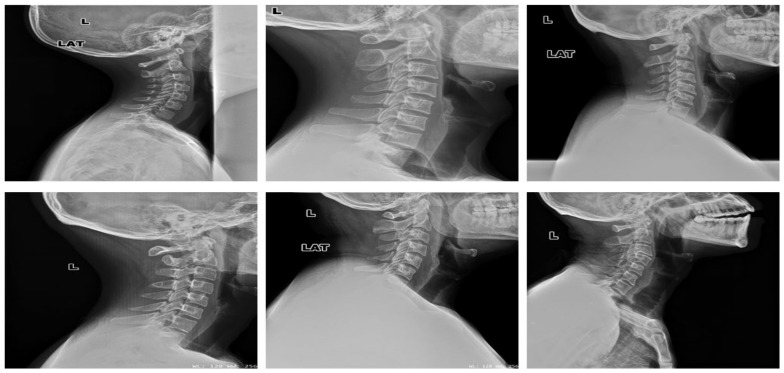
Samples from the third group (CS normal images).

The dataset has been obtained from Kaggle [36]. It contains 2009 images. The images are organized into three groups. The first group includes 530 CS dislocation images (Figure 6). The second group includes 772 CS fracture images (Figure 7). Finally, the third group includes 707 normal images (Figure 8).

### 3.2. Performance Matrix

The proposed method was trained on 70% of the dataset, validated by 15%, and tested on 15%. The proposed model’s performance was assessed using five quantitative measures (accuracy, sensitivity, specificity, precision, and F1-score) [37] from Equation (1) to Equation (5) and a qualitative measure called the ROC curve.

(1) $$\mathrm{Accuracy}=\frac{t_{p}+t_{n}}{t_{p}+f_{p}+f_{n}+t_{n}}$$

(2) $$\mathrm{Sensitivity}=\frac{t_{p}}{t_{p}+f_{n}}$$

(3) $$\mathrm{Specificity}\;=\;\frac{t_{n}}{f_{p}+t_{n}}$$

(4) $$\mathrm{Precision}\;=\;\frac{t_{p}}{t_{p}+f_{p}}$$

(5) $$F1-\mathrm{score}\;=\;2\times \frac{t_{p}}{t_{p}+f_{p}+f_{n}}$$

The symbols $f_{p},f_{n},{\;t}_{p},$ and $t_{n}$ are refer to the abbreviations of false positive, false negative, true positive, and true negative, respectively.

## 4. Results

The experimental results and analysis of the proposed method used in the dataset are presented in this section.

### 4.1. Results and Discussion

The proposed method was trained for 30 epochs with a batch size of 10. We used the SGD optimizer with an LR of 0.001 and a CCELF to calculate the validation error for each epoch’s training, the training error, and the validation accuracy. 

**Figure 9:**
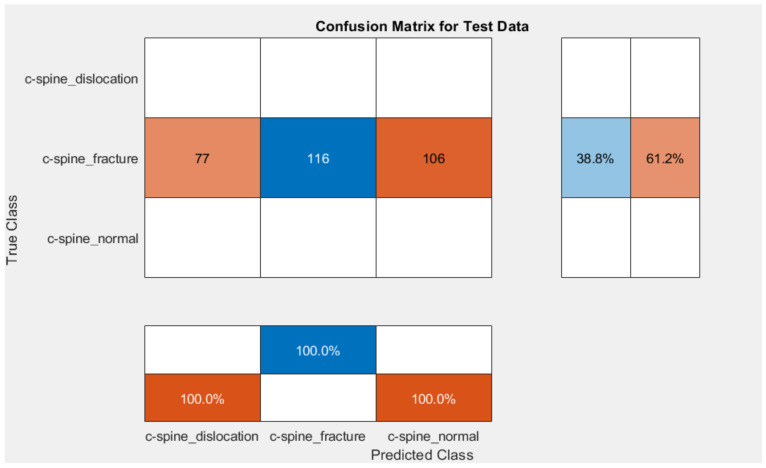
Confusion matrix for the proposed method using AlexNet.

**Figure 10:**
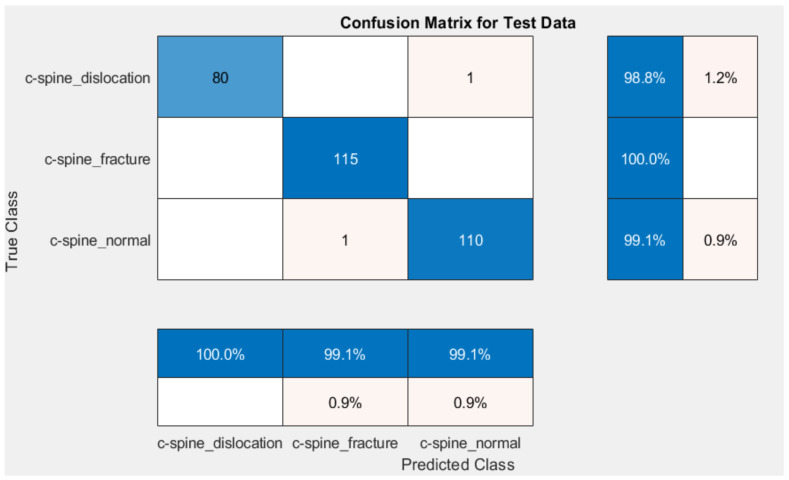
Confusion matrix for the proposed method using GoogleNet.

**Figure 11:**
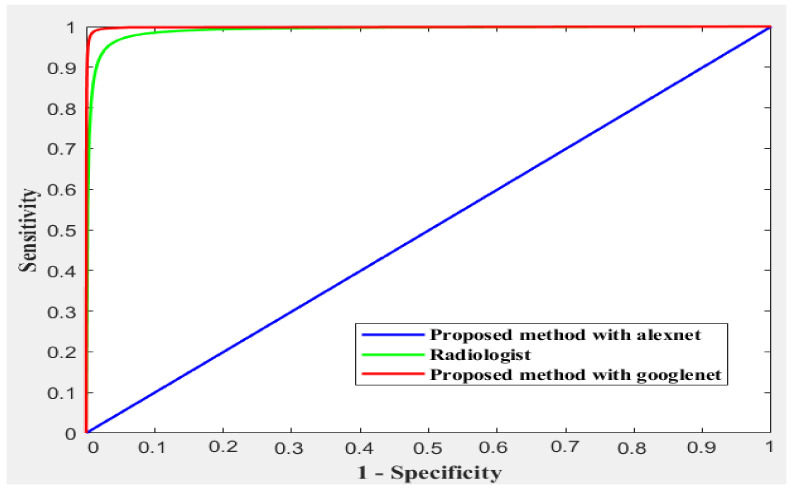
The ROC curves of the compared methods.

Different methods exist to update the deep learning parameter instead of using SGD only to update the network parameter. One common method is gradually lower the learning rates: starting with one learning rate for the first few rounds, switching to a different lower learning rate for the following few iterations, and lowering the learning rate even more for the following few iterations. An annealing method is used with SGD to accelerate the optimizer convergence and achieve minimum errors. Scheduled annealing is proposed with SGD to update the network parameters. This algorithm helps avoid local minima and saddle points and convergence to the global optimum solution is made possible by the scheduled annealing, which directly regulates the stochastic noise. In this study, we decreased the LR every four epochs to maintain the fast computation with a high LR to set up scheduling annealing with SGD. The confusion matrix for AlexNet and GoogleNet is shown in Figure 9 and Figure 10, respectively. The obtained results are summarized in Table 1. The ROC is shown in Figure 11.

#### 4.1.1. Comparative Study 

Using the same dataset, we compared the proposed method with the radiologist. The radiologist classification accuracy was 95% (95% CI, 94–97%), with 93% (95% CI, 88–97%) sensitivity and 96% (95% CI, 94–98%) specificity. The misclassified fractures by the CNN and radiologists are due to high similarity. They include the fractured anterior osteophytes, transverse processes, and spinous processes, as well as lower CS fractures often obscured by CT beam attenuation. Table 1 shows the result in quantitative percentages, while Figure 8 shows the ROC for different methods. 

Table 1 and Figure 10 compare the proposed method against the radiologist. The dataset contains a high fracture prevalence. Therefore, an evaluation of the obtained measures used the refined GoogleNet to detect CS fractures against radiologists. This choice was made since the group of interpreting radiologists in our study was diverse and included persons with different levels of competence in evaluating CS damage. The refined GoogleNet classification rate was 99.55% against 95% for radiologists. As shown in Table 1, the proposed method was the highest performance measure against radiologists.

From Table 1, the lowest measurements were for the proposed method with AlexNet, where AlexNet contains a limited number of layers and filters with each convolutional layer. The AlexNet layers are also directly connected to each other (serially). According to these results, AlexNet is prone to overfitting. Therefore, it fails to classify images belonging to classes containing fewer images. The proposed method based on GoogleNet performed best compared to AlexNet and the radiologist. GoogleNet contains different convolutional layers with different filter sizes. In addition to incepting the connection between layers, according to the architecture of the GoogleNet, it can also accurately extract features—even with a small dataset.

#### 4.1.2. Clinical Case Study

**Figure 12:**
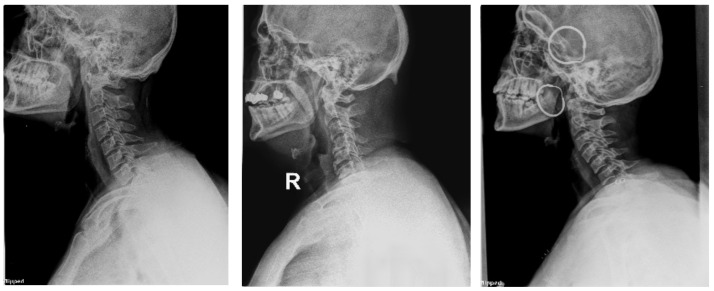
X-ray images for the normal CS.

**Figure 13:**
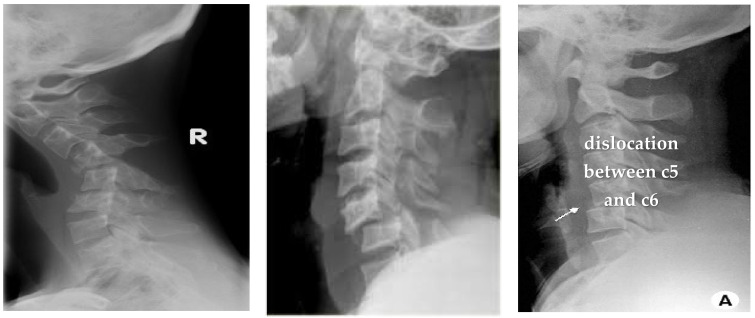
X-ray images for the dislocated CS.

**Figure 14:**
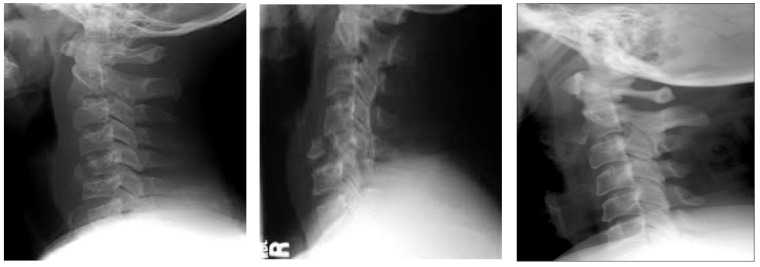
X-ray images for the fractured CS.

To evaluate and prove the ability of the proposed method to classify CS X-ray images in dislocation, fracture, and normal, we obtained 68 unlabeled CS X-ray images gathered from a radiology center. A team of orthopedic surgeons classified the clinical X-ray images into 61 normal, 3 dislocation, and 4 fracture X-ray images due to the absence of fracture features, fissure lines, vertebra compression, loss of vertebral height, or dislocation in the form of loss of the vertebral alignment or facet joint separation. Four fracture images due to the fracture lines and loss of bony continuity together with vertebral compression, and three dislocation X-ray images due to loss of vertebral alignment and facet joint separation Figure 12, Figure 13 and Figure 14 show a sample of normal, dislocated, and fractured CS images, respectively.

The proposed method of using GoogleNet was used to classify the clinical X-ray images against radiologists and orthopedic surgeons. Table 2 shows the accuracy rate of classifying the CS clinical images.

As shown in Table 2, the radiologist correctly identifies 66 images from 68 images. The wrong classification was in two normal X-ray images classified as fracture and dislocation. The orthopedic surgeon correctly identified 67 images from 68 images. The wrong classification was in one normal CS image classified as fractured. The diagnostic accuracy was lower because of the high variance in medical images and the difference in imaging parameters between the dataset used for training and the clinical real cases images. Small datasets might also be the reason for this. The use of more expansive databases and multiparametric CT could improve the diagnostic precision.

However, our study has certain shortcomings. The nature of DCNNs for the system was only given in images and the related diagnostic without precise descriptions of the features, which is a key drawback. One contradiction in DCNNs that deal with the processing of medical images is the “black box” mechanism; this is because the logic process used by these networks differs from that of people and computers. Due to people’s distinct logic processes, deep learning may leverage data qualities previously unknown to or ignored by humans (unless it is just a crude guess). Although our investigation produced encouraging results, the precise features employed are unknown.

#### 4.1.3. Saliency Map

**Figure 15:**
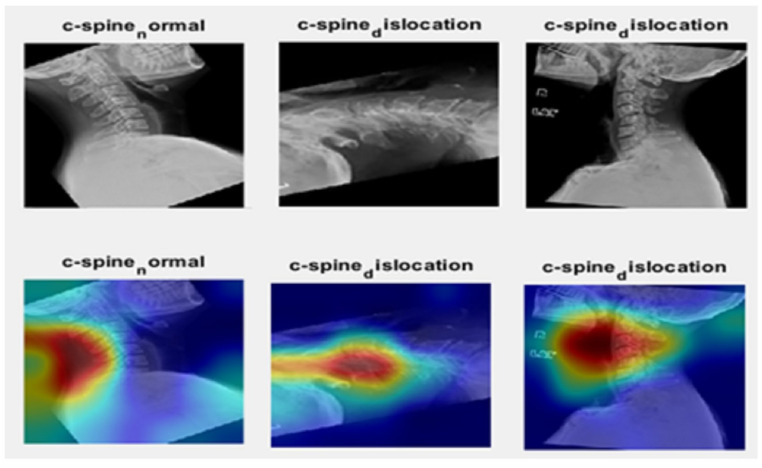
The first row is the image from the dataset with some rotation; the second row is the saliency map for the same images.

In this study, a saliency map was used to identify the image’s key areas and only analyzed those areas. We make some rotations to the image and used the proposed method to highlight the ROI of the spine. As shown in Figure 15, the proposed method can detect the ROI (spine).

## 5. Discussion

In our investigation, the CNN’s accuracy was 92.16% compared to radiologists’ accuracy of 97.1% and orthopedics’ surgeon accuracy of 98.5%, demonstrating the CNN’s potential for fracture identification, but perhaps with slightly less accuracy than them. However, the time between the acquisition of the image and the CNN analysis was much quicker than the time between the acquisition of the image and the completion of the radiologist report, highlighting the importance of the CNN in worklist priority. By reducing the time it takes to diagnose and treat unstable fractures, worklist prioritization has great potential to enhance patient outcomes. High-volume practices with even lengthier radiological interpretation delays would benefit more from this perk.

In this study, we proposed an automated computer-aided-diagnosis system based on deep learning for classifying cervical spine injuries into fractures and dislocations. It has been shown that the power of the deep learning technique can be used and provide fast and accurate solutions to the automation of medical image (X-ray) diagnosis. The proposed system can identify cervical-spine fractures and dislocations in X-ray images, which can help physicians quickly detect any fractures or dislocation in the CS, reducing the time, improving the quality of medical services, and reducing the physician’s stress. The results of the proposed system achieved an accuracy of 99.55%, a sensitivity of 99.33%, a specificity of 99.66%, and a precision of 99.33%. In addition, the proposed method successfully classifies real cases. In the future, in addition to the CS fracture detection, a system can be developed that can perform classification for the fracture types of CS. 

From a medical view, we believe that this study will be feasible in real life, especially in screening emergencies to detect fractured or dislocated CS cases. That will help inexperienced physicians and health providers. However, the clinical decision will depend on more detailed clinical examination and investigations, including further imaging modalities such as computerized imaging (CT), magnetic imaging radiology (MRI), and laboratory investigations.

This study has significant limitations related to the study design and selection bias, which reduce the generalizability of our findings. While only one primary site was used for the dataset scans, a prospective, multicenter trial must be conducted. Moreover, the prevalence of fractures in dataset imaging was significant, which reduced the number of clinically concealed fractures and may have unintentionally increased the sensitivity of our reported CNN and radiologist. These findings must be replicated in a dataset with a lower fracture prevalence, representing everyday clinical practice. Therefore, we see our findings as a crucial first step in proving CNN’s efficacy in detecting cervical spine fractures in a dataset with a high fracture prevalence and robust ground truth analysis.

## 6. Conclusions

The fracture or dislocation of the CS constitutes a medical emergency and may lead to permanent paralysis and even death. The accurate and rapid diagnosis of patients with suspected CS injuries is important to patient management. In this study, we proposed an automated computer-aided-diagnosis system based on deep learning for classifying CS injuries into fractures and dislocations. It has been shown that the power of the deep learning technique can be used and provide fast and accurate solutions to the automation of medical image (X-ray) diagnosing. The proposed system can identify CS fractures and dislocations in X-ray images, which helps physicians quickly detect any fractures or dislocation in the CS, reducing the time, improving the quality of medical service, and reducing the physician’s stress. The results of the proposed system achieved an accuracy of 99.55%, a sensitivity of 99.33%, a specificity of 99.66%, and a precision of 99.33%. In addition, the proposed method classifies real cases successfully. In the future, in addition to the CS fracture detection, a system can be developed that can perform classification for the fracture types of CS.

## Figures and Tables

**Table 1:** Quantitative comparison.

	Accuracy	Sensitivity	Specificity	Precision	F1-Score
Radiologist	95%	93%	96%	87%	
The proposed method with AlexNet	59.2	33.33	66.67	13.0	18.67
The proposed method with GoogleNet	99.55%	99.33%	99.66%	99.33%	99.67

**Table 2:** The accuracy rate of clinical CS X-ray image classification.

	No. of Correctly Recognized	No. of Wrongly Recognized
The proposed method	60 (92.16)	8 (1.84%)
Radiologist	66 (97.1%)	2 (2.9%)
Orthopedic Surgeon	67 (98.5%)	1 (1.5%)

## Data Availability

https://www.kaggle.com/datasets/pardonndlovu/chestpelviscspinescans, accessed on 5 October 2022.

## Abbreviations

AI	Artificial intelligence
ROI	Region of interest
CS	Cervical spine
CNN	Convolution neural networks
ReLU	Nonlinear rectified linear units
DCNN	Deep convolutional neural network
SGD	Stochastic gradient descent
LR	Learning rate
CCELF	Categorical cross-entropy loss function
ROC	Receiver operating characteristic
